# Advanced 3D Through-Si-Via and Solder Bumping Technology: A Review

**DOI:** 10.3390/ma16247652

**Published:** 2023-12-14

**Authors:** Ye Jin Jang, Ashutosh Sharma, Jae Pil Jung

**Affiliations:** 1 Department of Materials Science and Engineering, University of Seoul, Seoul 02504, Republic of Korea; yjjang@uos.ac.kr; 2 Department of Materials Science and Engineering, Ajou University, 206-Worldcup-ro, Yeongtong-gu, Gyeonggi-do, Suwon 16499, Republic of Korea

**Keywords:** three-dimensional packaging, through-Si-via, Cu pillar bonding, hybrid composite bonding, high entropy alloys

## Abstract

Three-dimensional (3D) packaging using through-Si-via (TSV) is a key technique for achieving high-density integration, high-speed connectivity, and for downsizing of electronic devices. This paper describes recent developments in TSV fabrication and bonding methods in advanced 3D electronic packaging. In particular, the authors have overviewed the recent progress in the fabrication of TSV, various etching and functional layers, and conductive filling of TSVs, as well as bonding materials such as low-temperature nano-modified solders, transient liquid phase (TLP) bonding, Cu pillars, composite hybrids, and bump-free bonding, as well as the role of emerging high entropy alloy (HEA) solders in 3D microelectronic packaging. This paper serves as a guideline enumerating the current developments in 3D packaging that allow Si semiconductors to deliver improved performance and power efficiency.

## 1. Introduction

The next electronic horizon is made possible in many ways by the sophisticated packaging technique of a 3D stacking of Si dies. Power supply, signal integrity, form factor reduction, and heat dissipation issues may all be addressed using stacked Si dies [1]. Three-dimensional TSV is a popular electronic packaging technology that offers significant benefits in terms of device performance, size, and cost in 3D packaging technology [2,3,4]. As optoelectronic interconnects transition to larger bandwidth devices like those with 1.6T or greater, the necessity of improved package design has become increasingly apparent [5]. In particular, the TSV is vital in semiconductor integration, which enables rapid signal transmission due to interconnected upper and lower-stacked Si chips [4,5]. The complementary metal-oxide semiconductor (CMOS) image sensor, dynamic random access memory, flash memory, system in package (SiP), system on chip (SoC), Internet of Things (IoT), 6G/5G networks, and high-performance computers (HPC) are various industries where TSV technology is a key player [6,7].

The most recent developments in digital electronics, including the IoT, artificial intelligence (AI), 5G networks, and HPC, have called for computers with exceptional speed and computing abilities [8,9]. Moore’s law predicts that transistor density per unit area in microchips would increase exponentially to meet these criteria, even as chip size decreases [10]. The technology for mass-producing 5 nm chips has already been developed by popular semiconductor manufacturers like Samsung and Taiwan Semiconductor and Manufacturing Company (TSMC) and is on track for future commercialization. Nevertheless, to meet 3D integration criteria, e.g., high I/O terminals, speedy signal, less power requirement, and efficient thermal dissipation, electronic manufacturers have already developed many 3D, 2.5D, and 5.5D packages [11,12].

The TSVs are the basis of 3D integration that uses vertical interlayer interconnects to stack and connect different functional chips. The X and Y dimensions are dramatically decreased while the Z dimension hardly changes. The system performance is improved through a large reduction in the connection length [13]. Thermal dissipation in 3D chips is challenging because the heat generated in the bottom chip must dissipate across the entire stack [13,14]. Functional chips are stacked vertically in a 2.5D packaging process using a silicon imposer to address this issue [15]. TSV technology is required for the vertical stacking of Si chips for high-density chip packing in semiconductor system integration. Systems integration may also make use of 3D packaging, which enables components to be linked over the lowest practical distance than 2D/2.5D platforms. Chiplet System Integration is one of the 3D packaging-based products that Intel and other companies have produced for use in AI and HPCs. According to a recent report by Samsung Electronics, Rogowski coil current sensors in micro-electro-mechanical systems (MEMS) can be produced by stacking 12 memory chips greater than 6 × 10^4^ TSVs [16,17,18,19,20,21,22].

Industries are investigating novel TSV filling technologies such as electroplated solder bumping (higher density than ball bumping and paste printing), ref. [23], hybrid bumping [24], nano-modified solder bumping [25], emerging HEA-based solder bumping [26], and bump-free technology [27]. Sakui et al. [27] created a wafer-on-wafer package that joins TSVs without any solder bumping. This method makes it possible to directly link bump-free TSVs between the top and lower Cu-fillings, resulting in a reduction in package thickness. A solid understanding of TSV and solder bumps is necessary to ensure the reliability of fine-pitch 3D chips. This review is concerned with how TSV and solder bumping affect joint reliability while constructing fine-pitch connectors. In light of the emerging solder bumping technology, composite TSV filling materials (Cu-X, X = metallic nanoparticles (NPs), carbon nanostructures (graphene, carbon nanotubes (CNTs)), etc.), this report also guides the reader towards the potential use of emerging HEAs in fine-pitch TSV and solder bumps in the future 3D packaging.

### 1.1. Moore’s Law

The ability to stack numerous chips together greatly boosts system integration and helps to fulfill the rising need for system performance (Higher than Moore) and functionality (Beyond Moore). Vertical continuity between neighboring stacked layers minimizes the global interconnect length, hence lowering the RC delay compared to traditional wire bonding [28]. The 3D chips provide several advantages, including better form factor, density, integration, cost-effectiveness, and performance, such as decreased power consumption and signal propagation latency [29]. Combining wire bonding [30] with TSV [31] is commonly utilized to exploit 3D integration practically. Deep reactive etched Si layer, dielectric insulation layer, super-conformal Cu-filling, and a metal-oxide-semiconductor (MOX) structure are the various parts in a high aspect ratio (HAR) TSVs [32]. TSVs offer vertical linkages between various layers in contrast to standard planar interconnects buried in the dielectric layer.

Nonetheless, owing to a differing coefficient of thermal expansion (CTE) and electrical properties of various layers, there are difficulties with mechanical stress [33] and electrical parasitic effects [34]. The TSV capacitance must be kept as low as feasible, and for this reason, using a low-dielectric liner might significantly decrease the CMOS backend capacitance and give various benefits for circuit performance [35]. When there is uneven heating that results in hotspots all over the chip, a steady TSV parasitic capacitance is also desirable. TSVs may be used as passive heat dissipation components incorporated into the 3D IC stacks because of the outstanding heat-dissipating capabilities of the Cu core and Si-chip. Thermal management poses a substantial issue for 3D technology as well. Nevertheless, the Si substrate experiences thermomechanical stress owing to a wide gap in the CTE of Cu and Si-wafer, which might potentially affect dependability. The electron/hole mobility of Si near the TSVs may also change because of this stress [36,37].

### 1.2. Evolution of Through-Hole Technology

Moore’s Law, developed in 1965, has been a major driving force behind the growth of the microelectronics industry for the past 50 years. After years of rapid scaling, lithography is already hitting its limitations, as observed recently [38]. The Si packaging efficiency consistently grows from 10 to 90 and 100%. Towards thinner interconnects, fan-out packaging where smaller dies are integrated may achieve higher production efficiency in future 3D packaging [39,40,41,42,43,44]. It is now commonly known that 3D integration makes it feasible to achieve high interconnect densities, wide bandwidth, minimum energy consumption and form factors, and the heterogeneous integration of numerous packages [45,46,47,48,49,50,51,52,53]. The superior bandwidth gained by the 3D integration of various memory devices has refueled interest in parallel and sequential HPC technologies, which are regaining popularity in cutting-edge fields like deep learning, neuromorphic computing, networks-on-chip, and 5G/6G communication networks [54,55,56,57,58].

### 1.3. State of Art Publications on TSV

In this review, a complete investigation of 3D TSV technology has been made with a focus on well-known die-stacking TSV and solder bumping methods. Recent advancements in TSV fabrication, TSV filling materials, functional layers, and bonding methods like Cu pillar bumping, ball mounting, paste printing, solder injection, electroplating, and bump-free joining have been overviewed to gain insight into their application in industries and related issues.

**Figure 1:**
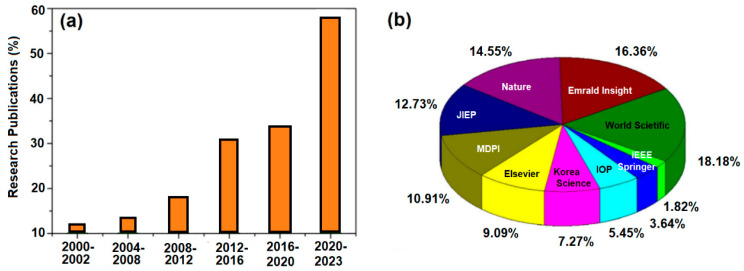
(**a**) Last decade growth in TSV research literature. (**b**) The breakdown of research articles obtained from different research databases [59].

Figure 1a displays the percentage of TSV articles amongst other fields that were published between 2000 and 2023, including theses and patents on TSVs related technologies. As a reference, the various articles published by different publishers in last decade according to Cho et al. [59] is also shown in the chart (Figure 1b).

The main goal of the study is to review recent progress in TSVs and 3D integration for future manufacturing. The fundamental manufacturing procedures for TSVs, including various functional layers for 3D integration, are briefly summarized in Section 2 of this article. Section 3 is devoted to the methods of filling of TSVs, and various issues associated with them. Section 4 discusses various bonding methods of TSVs. Section 5 and Section 6 are based on the various bumping methods in detail, such as solder bumping and Cu pillars. Section 7 is related to hybrid bonding. Section 8 summarizes various reliability issues in TSVs, future recommendations followed by a brief outline of nanomodified and emerging HEAs solder bumps. A summary and forecast are provided in Section 9 to conclude the study.

## 2. Fundamentals of 3D Integration Technologies

The fabrication processes utilized for 3D integration are briefly discussed in this part, with an emphasis on TSV and wafer bonding technologies. We also emphasize the innovations that are especially suitable for MEMS applications.

### 2.1. 3D Integration and 3D TSVs

TSVs are used in this configuration to electrically connect the various chips and circuits on either side when stacking several wafers one on top of the other. Bonded metal bumps connect the neighboring chips across the bonding layers. TSVs are through-chip conductors that connect two chip surfaces. The bonding layers are made of a dielectric (insulator) and a metal. The dielectric (polymers, SiO_2_, air gaps) separates the conductors (metals, Cu, W) from the substrate (Si). A typical TSV consists of successive layers, i.e., adhesion, barrier, and seed layer onto the insulating dielectric [59,60,61,62]. A substantial portion of the 3D stacking uses TSV fabrication and wafer bonding [63,64,65]. Important steps in this process include Deep reactive ion etching (DRIE) to etch deep vias in Si wafers; deposition of a dielectric (SiO_2_) on the sidewalls; sequential deposition of adhesion, barrier, and seed layers (Cu) onto the dielectric; TSV filling through Cu electroplating; fabricating redistribution layers (RDLs) and metal bumps and further stacking of Si wafers. Highly-doped Si and W are mostly used via filling materials due to their tolerance to high temperatures (>1000 °C) in these techniques [65].

### 2.2. TSV Sidewall with Scallop-Free Etching

**Figure 2:**
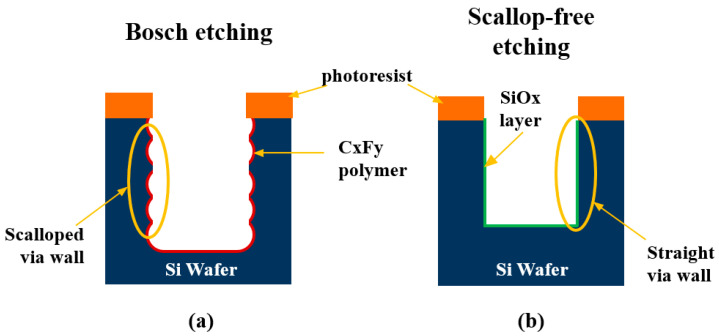
Schematic of TSV fabrication through Bosch process with scallop (**a**) and scallop-free etching process (**b**).

DRIE, metal-assisted chemical etching (MacEtch), and laser drilling methods have been used to fabricate TSVs in a silicon wafer. The DRIE process adopts plasma for anisotropic etching technology of Si and is the most widely used process, accounting for about 95% of the various methods of forming through-holes in silicon wafers [2,6]. The DRIE method includes Bosch and Cryogenic processes. The Bosch process is characterized by forming a “scallop” on the TSV sidewall (Figure 2a). The DRIE technique alternates between the etching and passivation stages to create through holes. Such repeated etch/deposit stages in DRIE lead to the periodic scallop formation on sidewalls in vias. The presence of a scallop can cause troubles such as copper ion diffusion from Cu-filled TSV to the inside of the SiO_2_ insulation layer and results in SiO_2_ failure and current leakage. This can lead to device performance degradation, and wiring reliability is reduced [66,67].

Several methods have been studied to control scallop formation in the TSV sidewall. The cryogenic process is one of the methods used to solve the “scallop” problem in the Bosch process, which involves supplying (O_2_ or CHF_3_ + C_4_F_8_)/SF_6_ gas and lowering the temperature of the substrate to −100 °C or less. However, the Cryogenic process requires a liquid nitrogen cooling device and increases the overall process time through the cooling/heating cycle [6,67,68]. MacEtch is also known to produce few or no scallops on the side walls, according to several studies [69,70,71]. This method can be performed in the laboratory without expensive equipment, allowing for the creation of a HAR structure [72]. The disadvantage of MacEtch is that metals used as catalysts, such as Ag, Au, Pt, Pd, and Cu, are expensive, and the diffusion of silicon can adversely affect the performance of CMOS, etc. [73,74]. Recently, the addition of TiN and various carbon nanomaterials such as CNTs, graphite, graphene, and graphene oxide (GO) have been reported [75]. It can also be applied to Si-free semiconductors such as Ge, GaAs, GaN, InP, and Ga_2_O_3_. Nuraini et al. presented deep etching micro-patterns in silicon using a new MacEtch method [76]. They used ultra-thin metal films to promote the “out-of-plane” mass transfer at the Cu/Si interface, which led to micrometer-wide holes. Deep vertical holes over 200 μm were etched at a high speed (>0.4 μm/min).

Murata et al. also investigated the MacEtch to evaluate the influence of etchant composition on the etching profile on a Si <100> wafer [77]. The etching rate increased with the molar ratio (*ρ* $=\left[HF\right]/[HF]+[H_{2}O_{2}]$), and Si-HAR structures were formed at a high etching rate (2.3 μm/min). Hanatani et al. have formed HAR vias on a Si substrate using the MacEtch process for the application of TSV holes [78]. The shape of the etching hole was improved from a bent to a straight shape by adding surfactants benzalkonium chloride (BKC) and polyethylene glycol (PEG). Nguyen et al. demonstrated the influence of SF_6_ flow rate on the sidewall shape [79]. When the flow rate of SF_6_ was 5, 10, and 15 sccm, the slower the flow rate, the deeper and more linear the etching appeared; that is, the TSV sidewall became straighter at 5 or 10 sccm than at 15 sccm. Morikawa has lately reported the “Scallop-free” (Non-Bosch) etching method in TSV using mixed gas plasma such as SF_6_ and O_2_ (Figure 2b) [80]. The smooth (scallop-free) sidewall can shorten the deposition process.

### 2.3. Plasma Dicing of Si-Wafer with TSVs

**Figure 3:**
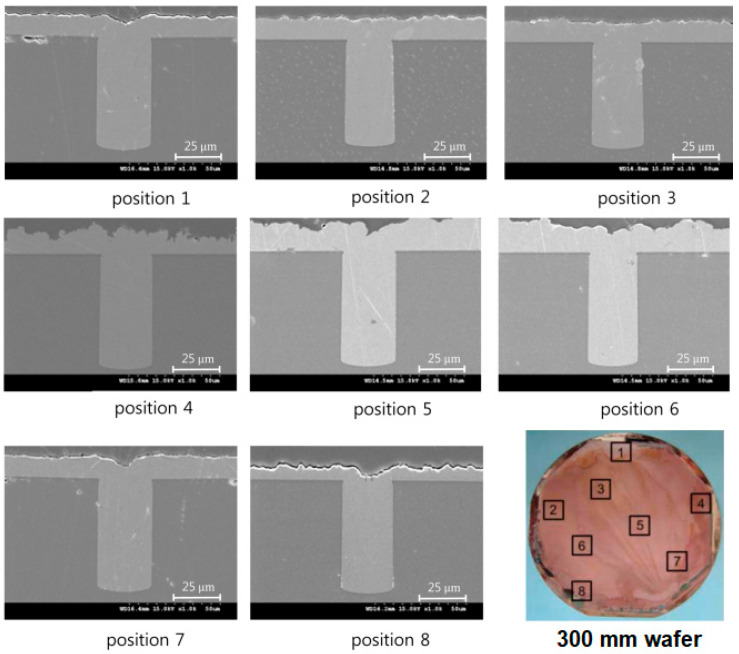
Plasma-diced Si-wafers. The cross-section images are shown with Cu-filling [80].

Plasma dicing produces fewer chippings after cutting and does not cause physical damage compared to mechanical or laser dicing. Thus, it demonstrates a better die quality and economy, which is suitable for dicing thin wafers [81,82,83]. Figure 3 shows a 300 mm plasma-diced silicon wafer plated with −7 to −10 mA/cm^2^ for 2.5 h [80]. The average silicon etching rate is 10 μm/min. Based on the electron microscopy observations, the uniformity of the Si-etching depth at nine locations exhibits good results with an error range of ±4.5%.

Westerman et al. have revealed that the die fabricated through plasma dicing had nine times higher die strength than dies that were cut mechanically [84]. In addition, 29,251 dies of 1 mm^2^ size were obtained from 200 mm diameter wafers using plasma dicing, showing a more efficient method than mechanical dicing (24,867 dies) and laser dicing (26,798 dies). A 775 µm thick Si wafer with a HAR of 13:1 and 20:1 has been attained utilizing plasma dicing [81]. They also showed how the process and sidewall quality for plasma dicing affected the die break strength at various orientations and presented a comparison of sidewall quality and process timings to traditional mechanical blade dicing for certain die sizes.

In order to improve etch features over TSV sidewalls, various plasma etch systems have been employed, such as inductively coupled plasma (ICP), capacitively coupled plasma (CCP), and electron cyclotron resonance (ECR) driven DRIE. High-density plasma formed at high frequency and high RF power in an inductor coil encircled by a dielectric is provided via ICP. Low damage is produced during ICP etching, while fast etch rates are maintained. ICP is available in a variety of configurations, including ferrite-core, planar-coil, and cylindrical-coil. An immersed ICP plus a traditional transformer-coupled plasma (TCP) placed at the chamber’s perimeter make up a planar ICP. In contrast, when reactive gases are introduced into the chamber, a plasma known as a capacitively connected plasma is created between the two electrodes. The plasma that results is referred to as CCP because the electrodes resemble the parallel plates of a capacitor. Whereas CCPs are usually utilized for dielectric etching, ICP plasma is usually employed for metal and conductor etching [85].

Because of the magnetic confinement of electrons in the source region, high-density ECR plasmas can form at low pressures with low plasma potentials and ion energies. Most people agree that ICP etching is superior to ECR in a number of ways, including simpler production scaling, better plasma homogeneity across a larger area, and reduced operating costs.

### 2.4. Functional Layers

A functional layer is a thin coating grown on the inner sidewall of the via before metal filling. The functional thin films are formed in the order of an insulating (dielectric), adhesion, and seed layer. According to previous studies, the SiO_2_/Ti/Cu structure is most commonly used as functional thin films [2,86,87]. Table 1 summarizes recent studies of functional thin films in TSV. Generally, SiO_2_ is used as an insulating layer, but in rare cases, Al_2_O_3_ can be selected. Most of the seed layer is copper, but sometimes Pt [88] or Ru [89] can be chosen. Recently, ultrasonic-assisted electroless Cu was plated to form the seed layer by Chen and Xiao [90,91]. This process can achieve a continuous and dense Cu-seed layer with high-efficiency deposition, better scallop-step coverage, and uniformity. The ultrasound helps Cu ions to diffuse into the vias more effectively and increases the reaction efficiency. The seed layer can be Au instead of Cu because of better oxidation prevention and chemical stability in the plating solution. The various trends in the development of functional layers of a TSV are summarized in Table 1.

Polymer liners on the sidewalls have been used as insulating layers to release the stress generated inside the vias [100,101,102,103,104]. Polymers have lower elastic moduli than SiO_2_, which can better relieve stresses caused by the CTE mismatch Cu, and lower dielectric constants than SiO_2_, which can reduce the TSV capacitance. Moreover, it has been demonstrated that Cu-filled TSVs can benefit over annular airgaps by lowering residual stresses for high-power devices [105,106,107]. Important adhesion and barrier layers are reported in the literature, i.e., Ti-TiN, Ti-TiW, and Ta-TaN in chip-scale technologies. Smaller and HAR TSVs require conformal metal coatings deposited by various atomic layer deposition and metal-organic CVD [108]. Previous reports have shown ALD-coated conformal Ru seed and TaN barrier layers in TSVs with fine diameters < 5 µm and aspect ratios > 25:1 [109]. Metal-organic CVD has also demonstrated the TiN barrier deposition in TSVs in HAR TSVs [110].

## 3. Conductive Material Filling in TSV

### 3.1. Cu-Electroplating

A TSV filling material should have high electrical conductivity and thermal reliability. Filling materials that can satisfy these requirements include Cu, W, poly-Si, solders, and so on. Copper electroplating is the most used method for filling conductive metal, as it is cheaper to process and facilitates mass-production [111]. However, careless copper electroplating can cause various defects in the copper filling [2]. A successful copper electroplating into a 4 × 50 µm^2^ TSV with an aspect ratio > 10:1 has recently been shown by Qiu et al. [112]. They used a TiN barrier layer followed by an electroless Co layer. The step coverage of the Co liner and conformal Cu seed layer were 97% and 75%, respectively, which enhanced the acidic Cu electroplating without gaps.

A TSV with diameters of 1.0 µm and 1.5 µm and aspect ratios of 7 and 6, respectively, was electroplated by Hwang et al. [113]. To increase the coverage of the barrier/seed layer steps, they used the TSV shape with scallop-free taper-ends and effectively filled the TSV with defect-free copper. Wang et al. added a single sulfonic acid derivative (3-(2-(4,5-dihydrothiazol-2-yl) disulfanyl)-propane-1-sulfonic acid) and observed void-free TSV filling and suppression of outgrowths as compared to existing commercial complex additives [114]. Typical parameters for various TSV technologies are given in Table 2.

Hwang et al. have studied the effect of bath agitation on TSV filling efficiency [117]. Without agitation, bottom-up Cu-filling was obtained by applying a higher current to 10 μm × 100 μm TSV. However, in 5 μm × 50 μm TSV, the Cu filling failed at a lower current without agitation. Moreover, 10 μm × 100 μm TSV showed a pinch-off defect with agitation at 1.0 A. Zhao et al. investigated the influence of current density on filling TSVs using electroplating [118]. The authors reported that a current density of 0.05–0.2 A/dm^2^ caused 100% via filling and prevented pinch-off defects. Kim et al. have reported a defect-free bottom-up filling of TSV (5 µm × 60 µm) with a pulse current of 3 mA/cm^2^ and a plating time of 900 s [119].

The influence of deposition current and time on the thickness can be seen through Faraday’s law (Equation (1)). The metal ions receive electrons from the cathode, which are then reduced and deposited, and the weight (W) of the plated metal ions is proportional to the current applied and the plating time.

W = Z × Q = (A/nF) × Q = (A/nF) × it(1)

(Q = electric charge (C), i = plating current (A), t = plating time (s), Z = electrochemical equivalent (g/C), F = Faraday constant (96,487 s.A/mol), n = number of electrons transferred [120]. During the creation of TSVs, conductive metals are inserted within the through after functional thin films have been deposited there [120]. Equation (2) illustrates how metal ions (like Cu^2+^) are deposited within the vias when they acquire electrons from the cathode during the electroplating process, which uses the Si wafer as the cathode. According to Faraday’s law, the quantity of metal deposited is proportional to the current flow (Equation (3)).

Cu^2+^ + 2e^−^ → Cu (2)

W = ZQ(3)

**Figure 4:**
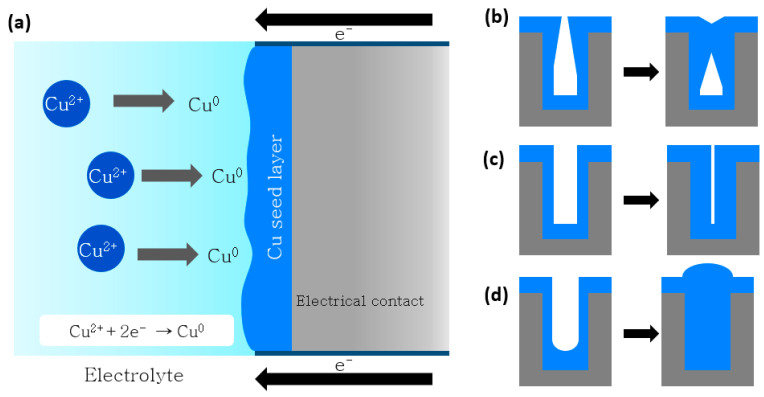
(**a**) Schematic of Cu deposition, (**b**–**d**) Subconformal, conformal, and superconformal filling profiles [126].

The schematic illustration of copper reduction at the interface is shown in Figure 4a. Optimization of plating additives and current waveform during electroplating results in the minimization of defects and improves filling efficiency. TSV defects, however, can result from an electric current concentration at the TSV’s entry corner during the electroplating process. Based on the geometry of the filled TSV, TSV filling may be divided into sub-conformal, conformal, and super-conformal filling [121,122,123]. The Cu electroplating and various classes of filling profiles are schematically shown in Figure 4a–d. Sub-conformal filling (Figure 4b) creates a gap within the TSV, whereas conformal filling (Figure 4c) produces a seam because the entrance corner plating moves more quickly. Super-conformal filling (Figure 4d) and faster plating from the TSV bottom are the only methods that will result in a flawless filling. The filling has been super-conformal, according to Lee et al. [123] and Hoffmann et al. [124]. Direct current (DC) filling of TSVs with conductive metals, such as copper, results in sub-conformal filling and the production of voids and seams at the entry corner of the TSV. Pulse current can be used to avoid these flaws. The early closing of the via entry is hampered by the need to alternate between the reduction current to fill the TSV and the oxidation current to dissolve any extra Cu on the surface. Setting the oxidation current to zero is an alternative strategy, although it can result in copper outgrowths due to unstable deposition rates [125,126].

### 3.2. Role of Additives and Process Conditions

**Figure 5:**
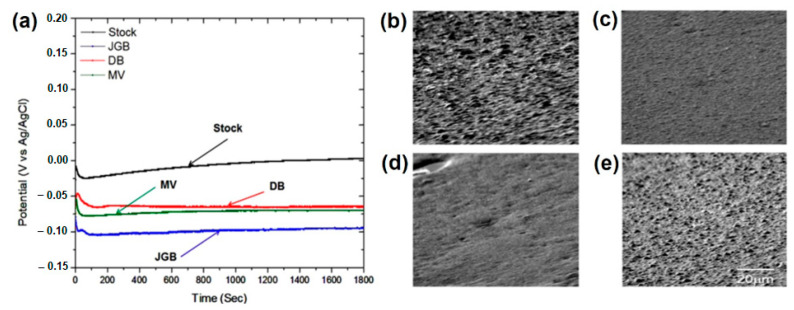
(**a**) Effect of various levelers on deposition potential [129], (**b**–**e**) corresponding morphology of Cu, (**b**) Stock solution, (**c**) JGB, (**d**) DB, and (**e**) MV [129].

The plating solution must comprise an accelerator, a suppressor, and levelers to use the pulse current procedure for filling TSV. Compared to bis-3-sodiumsulfopropyl disulfide (SPS), PEG often serves as a suppressor [127]. Different organic compounds such as levelers (Janus-Green B (JGB), di-azine black (DB), and methylene-violet (MV)) are used (Figure 5a) to fill TSVs perfectly. These levelers affect the current-potential kinetics and affect the Cu-deposition morphology in TSV [128]. The morphology of Cu filling material according to different chemical additives is investigated by Jung et al., as illustrated in Figure 5b–e [129].

Copper is not consistently plated in the absence of additives, as shown (Figure 5b). The surface morphology appears very rough, with nodular growth all over the cathode surface. In contrast, the surface morphology of Cu plated from JGB, DB, and MV was reasonably smooth and uniform (Figure 5c–e). Because levelers have a low concentration (50 ppm), they disperse and cling to the Cu surface well and improve further deposition [129]. These additives restrict the development of the plating layers as they adhere to the entry edge, enhancing the via filling quality. They are, therefore, regarded as inhibitors, whereas levelers are necessary to complete a perfect TSV filling. Several investigations have concentrated on altering the additives’ composition in the plating solution. The addition of PEG-PEG/SPS-I suppressor caused a decreased filling time by 50%, as shown by Kim et al. [130] by adding thiourea. Sung et al. [131] replaced the bromine ion in PEG-PEG/SPS-I with the I-ion to avoid the formation of unstable CuI, which reduced the filling time by 50%. Using the innovative leveler sulfonated-diallyl-ammonium bromide copolymer, Dinh et al. reduced the filling time by 5 min [132]. Similarly, Ha et al. [133] utilized sulfonated diallyl-dimethyl-ammonium chloride copolymer effectively to achieve a filling time of 3 min.

There have been several studies on different plating additives to optimize and enhance the TSV filling. For instance, Shin and his co-workers tuned the composition of multicomponent additives, i.e., PEG (suppressor), SPS (accelerator), and JBG (leveler), to achieve defect-free Cu filling in different aspect ratio TSVs [134]. Similar research has been conducted by Wu et al. [135], who investigated the interactions among different plating additives to obtain defect-free Cu filling. Tomie et al. [136] noticed that the levelers played an important role in building bottom-up Cu filling in TSVs even if the suppressors cover the Cu seed layer. The Cu-filling procedure was made simpler by Wang et al. [137] and Le et al. [138], as opposed to prior studies that utilized many chemicals simultaneously. Le et al. [138] proposed the use of 3-(1-pyridinio)-1-propanesulfonate (PPS) at a concentration of 5 g/L and a current density of 0.2 A/dm^2^ for defect-free TSV filling.

### 3.3. PPR Plating

The aforementioned discussion shows that it is difficult to find the optimum amounts of various additives in the plating bath for TSV filling. To overcome this concern, various methods have been developed to improve the plating speed using pulse reverse (PR) technology [139,140,141,142,143]. In general, copper deposition happens during the cathodic current cycle, and anodic dissolution takes place during the current-off cycle. The ions migrate to the favorable position, and layer growth occurs during the current-off cycle. However, perfect filling in TSV utilizing PR current is difficult, according to Lee et al. [123]. The authors recommended periodic pulse reverse (PPR) to improve the filling efficiency. PPR waveform contains a periodic variation of current, keeping the anodic cycle fixed. Nevertheless, the authors revealed that when the electrodes are 3 cm apart, the anodic and cathodic current densities were 30–50 mA/cm^2^ and 7–10 mA/cm^2^; sub-conformal fillings were achieved, which resulted in the formation of voids and seam defects. Likewise, Hong et al. [121] found that plating occurred near the mid portion of TSV, resulting in the creation of void and seam defects at a current density of 7.71 mA/cm^2^. This could be a result of the uneven bottom-up filling, which led to a Cu-seed point in the middle portion of TSV. Accordingly, Hong and his co-workers recommended a current-off delay between the cathodic and anodic current that lessened the via defects. Copper was plated during the reduction current, and overgrown layers were dissolved in the electrolyte during the oxidation cycle; with relaxation during the current-off phase, the dissolved Cu^2+^ ions diffused to the electrolyte. This is how the Cu grows in TSV when the PPR current waveform is used. This procedure aids in maintaining TSV open, preventing the development of voids and seams. Using a high-frequency and short-duty cycle PR waveform is another way to enhance Cu filling. This technique can encourage bottom-up filling and prevent outgrowths, as claimed by Jin et al. [144]. This study demonstrated that a TSV of size 6 × 60 µm^2^ could be filled in 30 min plated at 2 mA/cm^2^.

**Figure 6:**
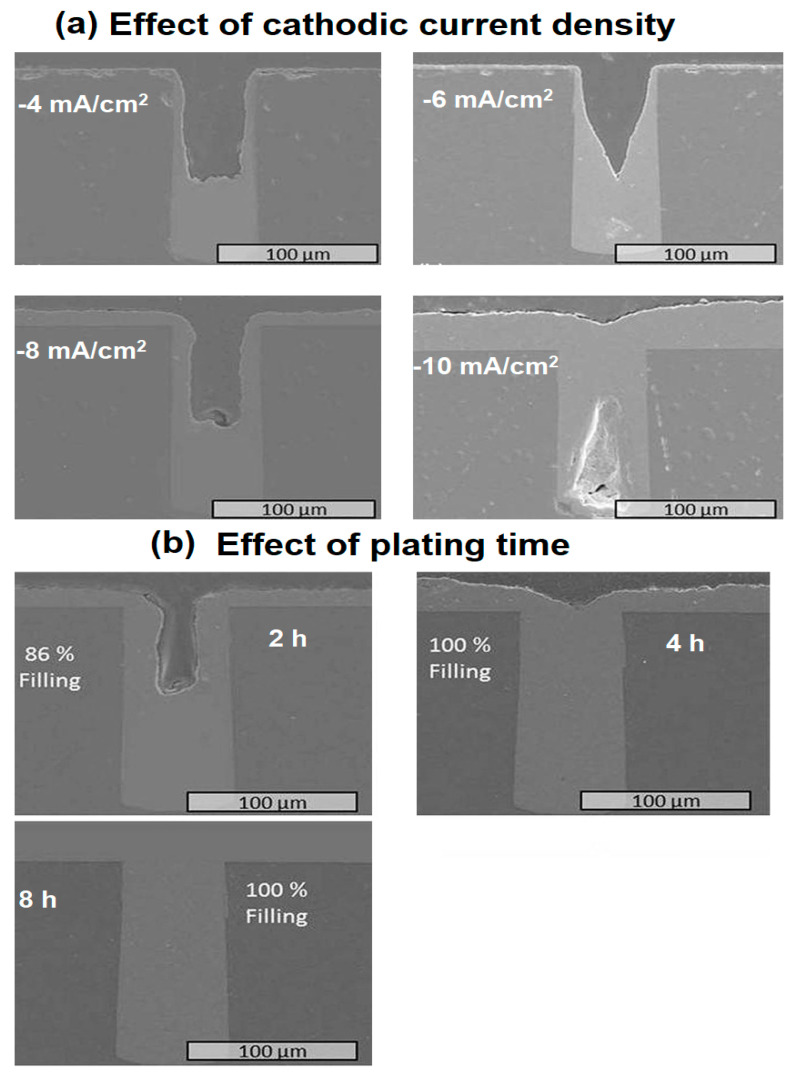
Effect of cathodic current density and time on TSV filling. (**a**) −4, −6, −8, −10 mA/cm^2^. (**b**) 2, 4, 8 h [145].

As the plating and etching phases occur at the reduction and oxidation currents, respectively, the pulse current plating technique is renowned for its prolonged filling time. Scientists have been trying to find a technique to speed up the filling process. Jung et al. investigated the effect of the filling time of TSVs (60 µm × 120 µm) using two-step PPR plating for low alpha solder bumping [145]. The authors found that the Cu layer grew with cathodic current and time, and a bottom-up filling was obtained (Figure 6a,b).

The Cu-filling in TSVs at various current densities of −4, −6, −8, and −10 mA/cm^2^ (anodic current density = 16 mA/cm^2^, plating time = 90 min). Inspection of Figure 6a shows that the filling tendency improves with increasing current density. The maximum filling tendency was achieved at −10 mA/cm^2^. The voids and cracks are introduced due to entrapped air at high current densities. The effect of plating time was further verified by fixing cathodic and anodic current densities at −8 and +16 mA/cm^2^, respectively. Figure 6b demonstrates the Cu filling tendency inside the via plated at 2, 4, and 8 h. The filling efficiency increased to 100% at 4 h using the optimized PR current parameters. Further, the authors also electroplated low alpha Sn-1 wt.% Ag-0.5 wt.% Cu (SAC105) microsolder bump of 83 µm reflowed at 245 °C for solder bumping applications.

Other research by Hong et al. [146] has shown that the filling time could be lowered by 1.36 times when using Cu-Ni alloy filling via PPR plating. Researchers have recently used ultrasonic vibration to increase the Cu filling rate and TSV filling speed. For instance, Xiao et al. [147] used 105 W ultrasonic for 5 h to fill TSV with a HAR (20 × 200 µm^2^) and obtained a filling ratio of 98.5%. Similar research was conducted by Wang et al. [148] on the effects of accelerator composition, current density, and ultrasonic waves on Cu filling. They improved the filling ratio by 23% with the use of ultrasonic technology, and they filled a 20 µm × 60 µm TSV in 3 h. Moreover, Zeng and his co-workers used pulse current and ultrasonic technology to fill a 20 × 60 µm^2^ TSV within 150 min without any noticeable defects [149].

### 3.4. Electrical Properties of TSV

As TSVs are employed in many forms depending on chip needs, it is crucial to look at the factors that might influence their electrical characteristics, such as their shape, stacking method, and arrangement structure [150,151]. The impedance values of several TSV forms, including cylinder, square, elliptical, and triangle shapes, were compared by Jeong et al. [152]. The authors measured the voltage by applying current through an unknown resistor and analyzing the results using a four-point probe measurement technique. The square TSV shape outperformed other forms in terms of electrical properties at high frequencies and impedance reduction. Due to its huge outside area and higher insulating layer, the quadrangular TSV form offers stronger signal shielding than other designs.

Park et al. [153] predicted the electrical properties of several stacked TSVs, their aspect ratio, and the sidewall thickness for a single Si substrate. The authors claimed that as the density of stacked TSVs increased, the total resistance and capacitance of the system also increased in proportion to the width and depth of the TSV. The total resistance after stacking could be decreased using HAR-TSVs, while the capacitance could be decreased by raising the via pitch and SiO_2_ layer thickness. Belaid et al. [154] developed a model to calculate the time-domain noise in 3D chips. The authors used inverse Laplace transform and chain matrices to demonstrate that horizontal connections and the type of I/O terminals affect the via noise coupling.

### 3.5. Reliability Studies on TSVs and Thermal Management

**Figure 7:**
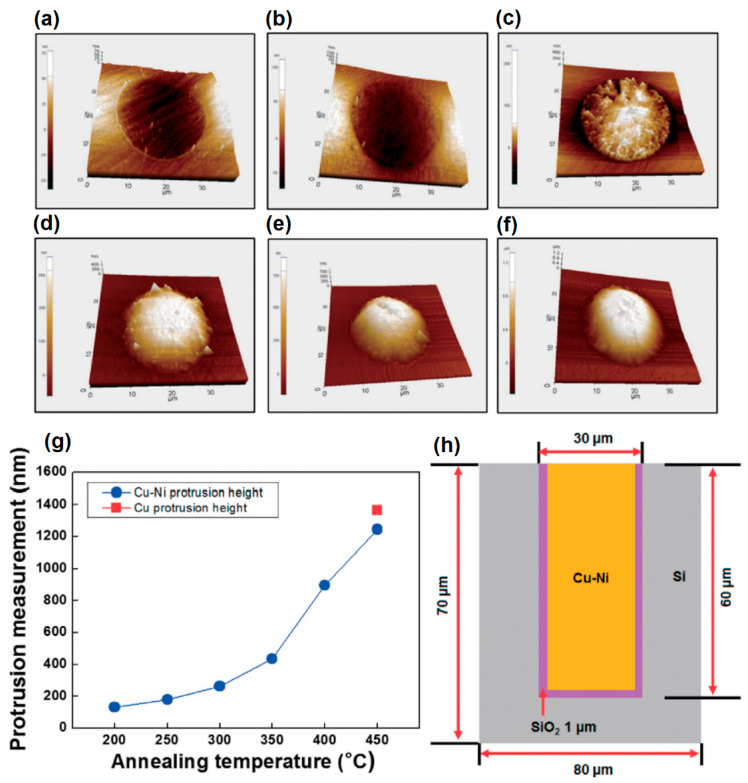
Surface topography of Cu-Ni filled TSV annealed at (**a**) 200 °C, (**b**) 250 °C, (**c**) 300 °C, (**d**) 350 °C, (**e**) 400 °C, and (**f**) 450 °C. (**g**) Measured extrusion height of Cu and Cu-Ni filled TSVs at different annealing temperatures and (**h**) Schematic of TSV structure used [156].

It is crucial to fill TSV with conductive material without any flaws, such as extrusions, voids, or seams (as illustrated in Figure 6), that affect the reliability of TSV to guarantee reliable interconnections at high thermal loading. The possibility of thermal stresses between the Cu-plated layer and the Si wafer caused by the CTE mismatch led to extruded growth of Cu at elevated temperatures. This may result in wafer warping and short-circuiting the entire 3D package [144]. As-plated Cu microstructure and plasticity behavior are two factors that affect extrusion failure. TSVs that go through thermal annealing after plating, however, behave linearly elastically without experiencing an increase in thermal stress [155]. The signal transmission in stacked chips may be hampered by the delamination of Cu from Si, but this problem may be resolved by lowering the TSV width or the Si/Cu CTE mismatch [155]. Different approaches have been suggested by certain researchers to control this issue. For instance, Jung et al. [156] demonstrated that replacing the Cu-filled TSV with a Cu-Ni alloy controlled the growth of Cu-protrusions during annealing. The surface topography of protruded Cu from the TSV structure after annealing at various temperatures is shown in Figure 7a–f.

The protruded height of the Cu/Cu-Ni filled TSVs was measured with 3D surface profile as shown in Figure 7g,h. After estimating the protrusion height with 3D surface profiles, the authors observed that the Cu-extruded growth was reduced significantly from 1360 to 1250 nm (an 8.8% reduction) when annealed at 450 °C. Roh et al. [65] electrodeposited Cu-W in tapered-shape TSV with entrance and bottom diameters of 44 µm and 34 µm and a height of 60 µm. The authors studied the extrusion characteristics of the Cu and Cu-W -filled TSVs. The results showed that the extrusion was suppressed by 44% after annealing at 450 °C in Cu-W flilled TSV compared to Cu-filled TSVs.

The extrusion-inhibiting effects of additives such as mercapto-benzimidazole sulfonic acid and thiourea at 400 °C were reported by Sung et al. [20] using TSV (5 µm × 60 µm). The aromatic structure of the material caused the 2M5S compound to diminish the Cu protrusion by 84.9%. The homogeneity of TSV was nevertheless decreased by the Cu (I)-2M5S compound that developed during plating. Thiourea, on the other hand, prevented Cu from protruding by 69.2%. Moreover, Jin et al. [144] showed that extrusion of Cu-filed TSV (6 µm × 60 µm) could be effectively decreased by applying a short pulse width and high frequency of 2222 Hz at 430 °C.

Recent researchers have focused on adding NPs in Cu-filling material to reduce the CTE mismatch and residual stresses between the Cu-filling and Si wafer. The CNTs have been used as a viable choice because of their excellent mechanical and electrical conductivity at the nanoscale [157]. Remarkably, compared to 72.9% for copper alone, the electrical resistance of a Cu-CNT composite is just 57.3%, according to Sable et al. [158]. In addition, the Cu-CNT-filled TSVs with graphene nanostructures served as better interconnects than pristine Cu. Their results showed that the interface resistance of CNT/graphene and Cu-CNT/graphene was reduced by 80.5% and 64.2%, respectively. The filling of the CNT-Cu composite in the TSV interposer was examined by Chen et al. [159]. The electrical conductivity (≈ 2.5 × 10^5^ S/cm) and the CTE (≈ 7 × 10^−6^/°C) values of the Cu-CNT filled TSVs were comparable to those of pure Cu and Si-wafers, respectively. Lwo et al. performed a Weibull analysis to analyze the performance of TSVs in harsh conditions [160]. They discovered that the durability of TSV was significantly impacted by thermal cycling and bias current. In biased samples, voids developed in the TSV corner, while oxidation, delamination, and flanking caused further reliability issues. Using finite element modeling, Jung et al. [152] investigated the Cu-filled TSV extrusion and stress distribution. They discovered that multilayer TSVs had greater fracture rates and worse reliability because of increased thermal stresses caused by the interaction amongst various layers. Moreover, they found that compared to elliptical and triangular TSV designs, cylindrical and quadrangular TSVs demonstrated superior mechanical performance. There are limited studies on TSV extrusion with the inclusion of NPs, even though elements affecting TSV reliability, e.g., via shape and filling materials, are documented already.

## 4. Bonding Methods in 3D Integration

Wafer bonding methods in 3D integration include dielectric and metal bonding [161,162]. Metal bonding binds metal bumps on two wafers to create electrical connections, despite dielectric bonding adhering to the wafers rather than the metal to boost the wafer strength. Whereas adhesive and fusion bonding are dielectric bonding methods, thermo-compression bonding (TCB), eutectic, and TLP are metal bonding methods [163].

### 4.1. Metal Bonding

TCB joins metal bumps via atomic diffusion in the solid state at temperatures typically between 200 and 400 °C. Enough pressure is applied to promote creep, shift grain boundaries, and increase diffusion for maintaining excellent contact at the interface. High temperatures and pressures cause metal atoms to diffuse over the interface barrier, which results in the formation of metal bonds and the successful joining of metals. Cu-Cu, Au-Au, Ti-Ti, and Al-Al bonding are just a few of the TCB approaches that have been used in 3D integration [164,165,166,167,168]. Among these, Cu-Cu bonding is most popular owing to its compatibility with TSVs, low process temperature, high strength, and improved electric conductivity. Cu-Cu bonding occurs effectively at room temperature (RT), while Al-Al bonding occurs at ~100 °C, as seen in the past [169]. TSVs may be directly used for Cu-Cu bonding, whereas microbumps are necessary for other metal bonding processes [170]. The development of eutectic systems of metals, on the other hand, entails a direct transition from solid to liquid or vice versa at a certain composition and temperature without going through a two-phase equilibrium [171]. When two metals are heated and brought into touch with one another, separate atoms create a combined super-lattice owing to atomic inter-diffusion. At eutectic temperature, the eutectic composition turns liquid, which, after solidification, joins the two metals. Eutectic bonding has been employed for hermetic packages using refractory metals such as Au-Si, Au-Sn, Au-Al, and Al-Ge [172,173,174,175,176,177].

In TLP bonding, an intermediate layer made of a low melting point metal layer is employed between two high melting contacts. The low-melting metal melts down when heated, resulting in faster solid-liquid diffusion and homogeneity of the liquid because of the higher atom mobility in the liquid phase. The interaction between the liquid and the solid melts joins the two together, forming intermetallic compounds (IMCs) after cooling. Temperatures are frequently raised by 20–30 °C above melting point to encourage proper bonding [178]. The advantage of TLP bonding is that it enables the joining of new layers without melting the current bonding metals during the succeeding bonding process since the resulting IMCs have a melting point greater than the process temperature.

Liquid-state bonding offers a high tolerance for topographical variations and surface oxides, as well as the ability to minimize stress and high yield at low pressure and temperatures. However, wafer sliding may impair the accuracy of post-bonding alignment due to thicker bumps in TLP bonding [179]. The most popular TLP material has historically been Cu-Sn owing to a low-temperature process (260 °C) and scalability [180,181,182,183]. Moreover, TLP bonding for 3D integration has utilized Au-Sn and Au-In [184,185,186].

### 4.2. Dielectric Bonding

Dielectric bonding techniques, such as polymer adhesive and SiO_2_ fusion bonding, are widely utilized to connect sections of chips. An interlayer of polymer serves as a bonding glue in adhesive bonding, which joins the chips together [160]. Epoxy [187,188,189], BCB (benzocyclobutene) [190,191,192], polyimide [193,194], and impression resist [195,196] are examples of thermosetting polymers that are often used as adhesives. BCB is a popular choice in 3D packaging because of its beneficial properties, which include strong bonding, less out-gassing, good chemical and thermal stability, and high bonding strength [187,188,189,190,191,192,193,194,195,196,197,198,199]. Adhesive bonding often requires high temperatures (frequently between 100 and 300 °C) and substantial bonding pressures to force adhesives to flow, taking into account variations in wafer thickness while easing the restrictions on surface topography. Wafer sliding, however, can result in severe post-bonding alignment problems because of the flowability of adhesives.

Fusion, or direct bonding, joins the wafers at RT, followed by high-temperature annealing to produce fine SiO_2_ layers on the Si substrate [200,201]. After wet/plasma etching, the SiO_2_ surfaces chemisorb H-O-H molecules and produce Si-O-H groups. Most of the Si-O-H groups polymerize at RT by creating (Si-O-Si) groups, which bond the two Si substrates strongly during annealing at 180–200 °C. The wafers must be very flat and smooth, with roughness < 0.5 nm and warpage < 20 µm, to achieve defect-free contact across large areas [202]. To provide mechanical support, typical polymer layers include acrylics, thermoplastics, polyimides, and elastomers [203]. Nowadays, polymers that can resist temperatures of up to 250–300 °C are occupied in the market, and efforts are currently being made to produce materials that can tolerate even higher temperatures [204,205,206,207].

### 4.3. Hybrid Bonding

Wafer joining and electrical connection are made possible by hybrid bonding that combines metal and dielectric bonding. Cu/SiO_2_ or Cu/adhesive bonding are the two most frequently used hybrid bonding methods [208,209]. Cu bumps and SiO_2_ layers are joined by RT bonding and subsequent annealing at 200–400 °C. Before bonding, CMP is necessary to planarize the SiO_2_ layers and Cu bumps [210,211,212]. The dish effect of Cu CMP [213], which causes the Cu to retreat at lower regions, limits RT bonding to the SiO_2_-SiO_2_ interface. The SiO_2_-SiO_2_ bonding is improved by the following annealing, which also brings the recessed Cu bumps into touch and bonds them due to their substantial thermal expansion. The industry makes widespread use of the resultant hybrid bonding method created by Ziptronix and marketed as DBI [214]. An important benefit of Cu/SiO_2_ bonding is high-density, good alignment, and bump-free bonding [215].

Cu/BCB adhesive bonding is more convenient to perform than Cu/SiO_2_ bonding because the adhesive deforms and relieves the surface topography. Partially cured BCB can be reflowed at high temperatures to adjust for surface projections between Cu and BCB [216]. As high temperatures might degrade BCB, hybrid bonding should be performed at moderate (about 200–250 °C) temperatures, followed by curing at 300–350 °C.

Low-temperature bonding is also achievable in TLP and adhesive bonding of low-temperature, Cu-Sn/BCB couple [217,218]. The liquid-state Sn may flex under the applied pressure during the Cu-Sn/BCB bonding process, resulting in a narrower and lower metal bump [217]. It is necessary to develop bonding adhesives before using BCB or polyimide for hybrid bonding. One can use dry etching, photopolymer adhesives [219], or spin coating and polishing [220]. An alternative method for creating adhesive layers involves applying dry films or laminating insulator underfill onto wafers before bonding [221,222]. At the proper temperature and pressure, the Cu bumps maintain their rigidity, whereas the dry films become softer during bonding. Direct Cu-Cu contact and bonding are made possible because of the pushing out of the dry films that were previously present between two Cu bumps.

## 5. Solder Bumping

**Figure 8:**
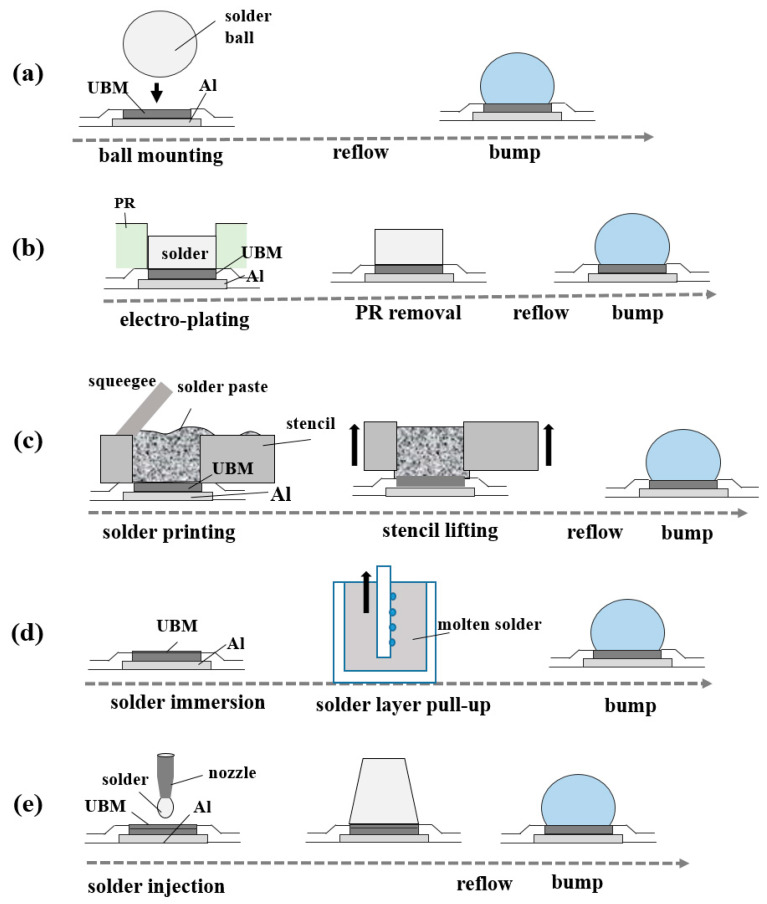
Solder bumping methods for interconnection: (**a**) ball bumping, (**b**) electroplating, (**c**) paste printing, (**d**) dipping, and (**e**) jet injection.

Bumps in Si-dies can be formed by various materials such as Sn-based solders, Au, Cu/Ni, and Ag; however, solder is the popular choice for high-performance semiconductor packages like 2.5D and 3D TSV [223,224]. Sn-based solders have excellent electrical conductivity and heat emission capability that enable the maintenance of high-density interconnection [225]. The various bumping methods (ball mounting, electroplating, solder paste printing, solder immersion, and solder injection) are schematically illustrated in Figure 8a–e. Among various bumping methods, electroplating is commonly used, which is almost 80% of the bumped wafers because it is inexpensive, easy for mass production, and creates fine bumps uniformly [226,227,228].

Yusuf et al. studied the effect of various solder compositions (Sn-40Pb, Sn-3.5Ag, SAC105, SAC305, etc.), surface tensions, densities, and geometry of solder bumps [229]. The range of solder density was 7.44 to 8.40 g/cm^3^, and that of the surface tension was 475–548 mN/m. According to statistical analysis using Statistical Package for the Social Sciences by IBM, the Pearson correlation coefficient (PCC) between the maximum bump width and the surface tension was −0.643, which indicates a close relationship. In addition, the PCC between the maximum standoff and solder density was −0.631, indicating an excellent match with the experiment.

Kim et al. have evaluated the microstructure and reliability of micro-bumps in TSV packages under high temperature aging at 125 °C, 150 °C, and 180 °C with different under bump metallurgies (UBMs) of Cu/Ni/Sn (solder), Ni/Sn, Ni/Cu/Sn [230]. No failure was seen in the TSV samples until 192 h at 180 °C for the Cu/Ni/Sn and Ni/solder samples, and their calculation showed aging at 150 °C for 1008 h was entirely safe. The IMCs are formed during soldering and under the service temperature. The IMC is formed between the Sn layer and UBM, which grows according to the surrounding conditions. IMC can have negative effects on void formation, wetting, reduced fatigue life, and tensile strength, so it is desirable to suppress IMC growth. In addition, previous studies have shown that the solder joint reliability decreases as the Cu_6_Sn_5_ IMC layer overgrows [72,231,232].

Table 3 shows the activation energies (Q) summarized from previous studies to form IMCs in solders. For Sn-3.5Ag, the activation energy is 65.9 kJ/mol, and for Sn-3.5Ag-0.007GNS, it is 77 kJ/mol [233]. Additionally, Sn-3Ag-0.5Cu and Sn-3Ag-0.5Cu-0.1 nano-TiO_2_ have Q of 57.71 kJ/mol and 69.32 kJ/mol, respectively [234]. In these studies, the addition of NPs increased the Q of IMC formation. According to Guo et al., NPs generally suppress the growth of IMCs in the solder [235]. The larger the Q value, the slower the reaction between Sn and Cu atoms and the more suppressed IMC growth.

Si chips are being incorporated more and more in the big data era to meet the rapidly expanding user demands. The development of distant learning and home offices has also significantly raised the demand for innovative electronic packages in the post-COVID-19 virus age. As microelectronic packaging in 3D integration requires a hierarchy of solders, low-melting solders must be developed to increase processing tolerance and create a broader processing window. Corresponding to this, low melting point lead-free solders have drawn growing technological interest in recent years [245]. High-temperature soldering increases the risk that the electrical devices being joined are susceptible to heat damage [246]. Low-temperature solders have also been prescribed for solder bumping applications in 3D packaging. For instance, flexible electronics, diodes, and liquid crystal displays require soldering at temperatures lower than 183 °C (melting point of Sn-Pb solder).

The eutectic points of Sn-Bi and Sn-In are lower than those of Sn-Pb and SAC 305 solder, which have eutectic points at 183 °C and 221 °C, respectively. The Sn-Bi solder is too brittle, and the Sn-In solder is ductile and has poor fatigue behavior [247]. Numerous research investigations have been conducted in this manner. According to the alloying additions, Sn-Bi-X-based alloys have a wide range of melting points. A thorough analysis of melting points and wetting properties is needed to identify appropriate applications. The melting point of Sn-Bi solders is affected by several metallic additives, including Cu, Ag, Ti, Sb, In, Zn, Co, Ce, and La, as well as oxide NPs (CNT, Y_2_O_3_, Al_2_O_3_, ZrO_2_, TiO_2_, Cu, and Ni) have been added [248,249,250,251,252,253]. The various compositions of low-melting Sn-Bi and Sn-Bi-X and their melting characteristics are summarized in Table 4.

The eutectic Sn-58Bi solder shows a melting point of 139 °C in the phase diagram [254]. According to the National Institute of Standards and Technology (NIST), the eutectic composition is Sn-56.97 wt.% Bi, and ASM International classifies it as Sn-57 wt.% Bi at 139 °C in the phase diagram. Nevertheless, Sn-58 wt.% Bi within a melting temperature range of 138–139 °C is also considered to be eutectic composition in numerous articles [254,255,256,257,258,259,260,261,262]. Eutectic Sn-In solder, on the other hand, exhibits exceptional softness and ductility. Eutectic Sn-In runs at a relatively high homologous temperature due to its 120 °C melting point, making its low creep resistance problematic [246]. Only minimally and with restrictions can the qualities of these two alloys be improved by the inclusion of a third element. The Sn-Bi-In ternary alloys are extremely complex, and as of yet, no organized study has conclusive findings [258,261]. The In32.5 wt.% Bi16.5 wt.% Sn solder, with an extremely low melting temperature of about 59 °C, has received the most attention [265]. Due to its low melting temperature and the fact that it is neither excessively brittle nor too ductile, solder can be used in flexible electronics. The limitations of the Sn-Bi-X and Sn-In-X solders are mostly understood. However, additional studies on ternary and quaternary solders should be conducted in the future.

## 6. Cu Pillars

**Figure 9:**
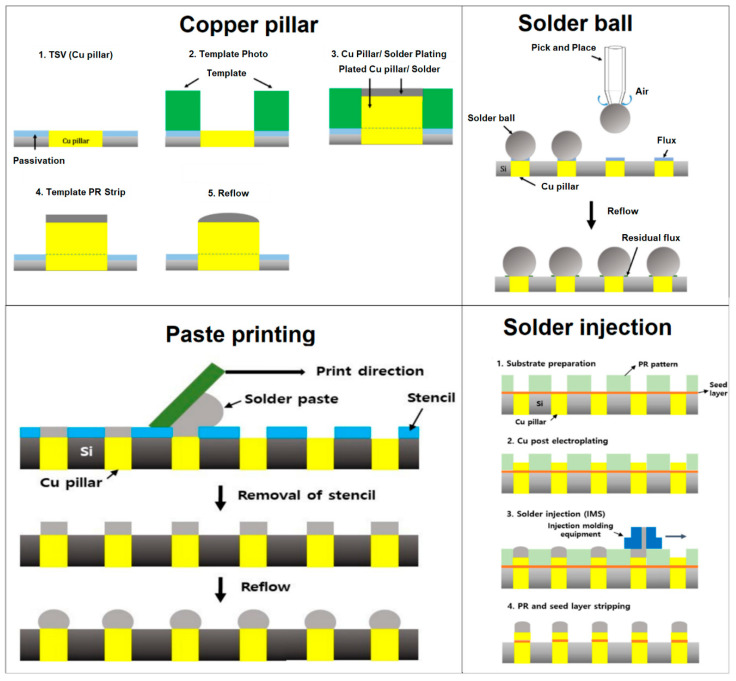
Schematic comparison of Cu pillar bonding with paste printing, ball mounting, and solder injection on TSV [59].

According to the trend of the smaller bump size and finer pitch, the pitch of the first-generation bump, more than 130 μm, has reduced to less than 30 μm in the recent third generation [266]. However, decreasing the bump size makes the underfilling process difficult. A Cu pillar bump (CPB) can provide a solution to this problem. The CPB is a cylindrical copper column instead of a conventional solder bump that connects a silicon chip to a substrate, and sometimes, the CPB has a solder cap. We have compared the Cu pillar bump with other solder bumping methods (ball mounting, solder paste printing, solder injection) in Figure 9. We can see that it is easy to adjust the gap between a Si-die and a substrate by changing the height of the CPB column, and the CPB improves contact resistance [226,267]. This direct chip connection method is referred to as a Chip Connection (C2) method [268] and is used for high density, fine pitches [269], and 3D chip stacking [223].

In an acidic electroplating solution made up of SnSO_4_, H_2_SO_4_, Ag_2_SO_4_, thiourea, and a few additives, Sharma and his co-workers effectively plated Sn-Ag bumps on the Cu-filled TSVs [21]. The authors used 40 min of plating time at the current density of 55 mA/cm^2^ to achieve eutectic composition. Further, the solder bump prepared was spherical and had the dimensions 35 µm × 46 µm. The solder bump was reflowed, and the joint shear test was measured. The results showed that shear strength increased by 35 MPa when the shear rate increased to 10 mm/s. A brittle IMC fracture mode was produced at higher shear speeds, which reduced the joint shear strength.

As shown in the Cu pillar method, a solder cap is formed onto the Cu pillar to join every Cu-filled TSV physically and electrically to stacked TSVs. Si is often electroplated using a photoresist and lithography to regulate the bump size precisely [270]. Tanida et al. [223] used a photoresist mold to create a small bump with a fine pitch. It could also be used to retain a space between the Si chip and prevent solder bridging of the Cu pillar. On the Cu-TSV, Sn- and Sn-Ag bumps were created using this method [271].

The pick and place procedure is used to micro-ball bump Sn-Ag and SAC305 solder on the TSV. Solder ball bumping is the most economical method that allows different solder bump sizes and solder balls. A high-precision assembly setup is necessary for choosing and arranging the micro-bumps on a fine-pitch Si chip. Using a Cu-filled TSV (60 µm × 120 µm), Jung et al. [145] demonstrated low alpha Sn-Ag solder ball bumping. The Cu-filled TSV was covered with a low-alpha SAC105 solder ball, which was reflown for 10 s at 245 °C peak temperature. In high-density electronic packaging, low alpha solder is useful for preventing soft errors. Numerous Cu_6_Sn_5_ IMCs were generated after reflow at the Cu/TSV interface. The IMCs thickness increased from 1.71 to 4.05 µm after aging at 85 °C/150 h.

The procedure of injecting solder onto a copper pillar is also known as injection molded solder (IMS). The tip on the Cu pillar is used in this procedure to force out molten solder metal [272,273]. The solder injection technique has the benefit of making ternary system solders like Sn-Ag-Cu. Unlike electroplating, the procedure is straightforward, and the bump geometry is independent of the placement on the wafer. Moreover, solder bumps of different sizes may be created, and the process is flux-free. A micro-bump is created via paste printing on TSV using a fine-pitch stencil and reflow. Several factors, including substrate surface quality, substrate-stencil ratio (pitch size, thickness, and gap), print speed, and pressure, must be carefully considered to execute paste printing properly and generate solder bumps [274].

In ultra-fine pitch TSV applications, paste printing is typically avoided due to poor print density. The solder paste-printing procedure has been made easier with current laser edge technologies and type-7 solder paste (size distribution: 2–11 µm). Moreover, paste printing provides advantages like cost-effectiveness and a simple process. Kim et al. printed Cu pillars on a flip-chip package using type 5 and type 7 solder pastes with a metal mask (30 µm × 50 µm) with an opening ratio of 70% [275]. The results showed that suitable printing could be accomplished with type-5 (10–25 µm) and type-7 (2–11 µm) solder pastes. Moreover, when the mask thickness was lowered to 30 µm (opening ratio of 100%), only type 7 solder paste was found appropriate for printing. The paste-printing method was also used by Kumar et al. [276] to produce a chip scale bump using type 7 solder paste. The authors used Ni-Co stencil with a pitch size of 120 µm and an opening size of 30 µm to form a solder bump of 100 µm. The design of the experiment method was applied to determine the ideal printing parameters. It was discovered that 7 kgf and 20 mm/s were the ideal squeezing pressure and print speed for defect-free bumping.

Recently, Xu et al. have performed an electromigration test using a copper pillar with Sn-1.8Ag solder and a current density of 1.5 × 10^4^ A/cm^2^ at 125 °C [270]. During electromigration, a large number of IMCs (mainly Cu_6_Sn_5_ and Cu_3_Sn) were accumulated at the anode. In addition, Kirkendall voids were formed in the cathode due to vacancy flux. Sn-grain with large α (α: angle between the *c*-axis of the Sn-grain and the electrons flow) showed excellent electro-migration resistance by delaying Cu diffusion and IMC accumulation. Li et al. have investigated the thermomechanical reliability of a flip-chip device using a Cu pillar/solder joint [277]. Individual thermal cycle tests were performed for the Cu pillar/Sn-Ag/Cu pad Cu pillar/IMC/Cu pad joints using TCB. Fatigue cracks in the Sn-Ag solder joint were generated at the corner of the Cu pillar and propagated along the micropores in the solder. The Cu pillar/IMC/Cu pad joint showed a lower lifetime than the Sn-Ag joint due to the presence of cracks at the interface of Cu_6_Sn_5_ and Cu_3_Sn IMCs.

### 6.1. Low-Temperature TLP Cu Pillar Bonding

**Figure 10:**
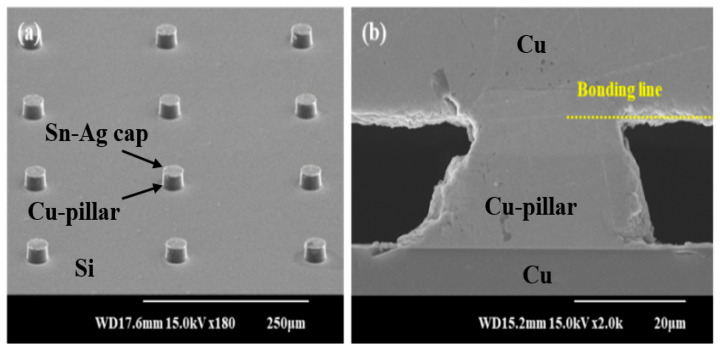

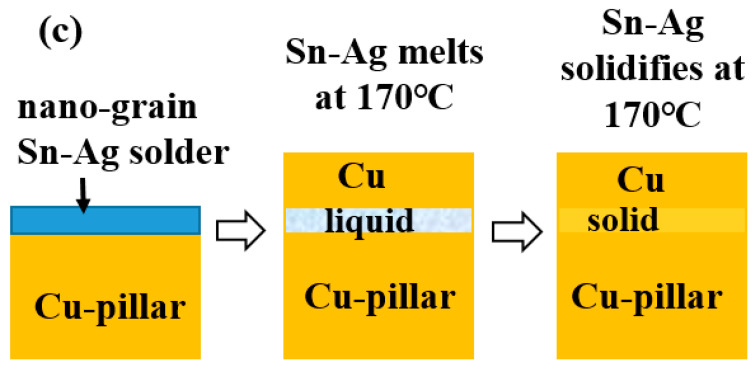
Cu pillar bonding with nano-grained solder at 170 °C. (**a**) before bonding; (**b**) after bonding; (**c**) low-temperature bonding procedure.

A low-temperature TLP bonding for Cu pillar using a novel nano-grained solder layer was performed in our previous research [271]. As shown in Figure 10a, the Cu pillar with a diameter and height of 30 μm and 15.5 μm was joined with a nano-grained Sn-Ag solder (5 μm) on the top of the Cu pillar (Figure 10a). Generally, the melting point of a metal powder with several nanometers in size is significantly lower than that of one with a size over micrometers. In the case of Figure 10b, the melting point of the nano-grained Sn-Ag solder was lowered to 140–150 °C, and Cu pillar/Sn-Ag/Cu was reflow soldered at 170 °C. Then, the melted solder solidified at 170 °C through isothermal solidification (i.e., the liquid solder transformed to solid at the same temperature of 170 °C), which is called TLP bonding. The eutectic temperature of a general Sn-Ag solder is 221 °C, and the normal soldering temperature is around 240–250 °C. Thus, bonding at 170 °C Cu pillar/Sn-Ag/Cu is a fairly low temperature. In the joint, the atoms in the solder melt diffuse towards the Cu pillar and Cu-pad, and Cu atoms diffuse from the pillar to the solder until isothermal solidification is complete. Consequently, the solder joints of Figure 10b,c show different solidified microstructures from the existing non-TLP bonding with a eutectic structure. After bonding, the melting point of the TLP joint increases due to the change in eutectic microstructure, and thus, the TLP joint has excellent properties at high temperatures where it does not re-melt at the bonding temperature.

### 6.2. Bump-Free Stacking of TSV

The newest way of joining for 3D connection is bump-free joining. The package-on-package connection technique, which uses TSVs without bumps, was initially introduced by Sakui et al. [27]. Bump-free TSVs may be attached between the top and bottom Cu pillars to thin down the package. The authors have reported a thickness of 600 µm for bumped chips and 60 µm for bump-free connections in a 3D stacked system of TSVs with six dies for a multicore central processing unit (CPU). Hence, a factor of 10 is taken off the die thickness. Bump-free joining indicated a temperature gain of 5.8 °C when eight stacked dies with solder bumps on TSVs were compared. Bump-free technology is used instead of conventional TSVs with micro-bumps that experience temperature rises, allowing for the creation of bump-free stacking of TSVs in HBM (High Bandwidth Memory) with eight dies. The authors also suggested using bump-free technology to create an object with a volume of 50 mm^3^ and a consumption of 0.5 mW in modern AI robots containing a CPU, ultra-small business, HBM, and memory sensors.

## 7. Hybrid-Bonding of Cu and SiO_2_

Among various Cu bonding methods for implementing 3D packaging technology, there are surface-activated bonding (SAB) [278], self-assembled monolayer (SAM) [279], nt (nanotwinned)-Cu [280], metal passivation [281], and hybrid bonding [282]. Of these, hybrid bonding technology is receiving a lot of attention as the core of next-generation 3D IC technology [283,284]. Hybrid bonding is a bump-free bonding method that combines a metal with an oxide or a metal with a polymer and physically and electrically connects two substrates at the same time [285,286]. In addition, fine pitch bonding can be achieved without solder-capped micro-bumps, which improves bandwidth and power efficiency due to more interconnects. The existing bonding process has low processing performance, and scaling to a pitch smaller than 40 μm is difficult [85]. Thus, to achieve the ultrafine pitch required for future high-performance devices, hybrid bonding is essential [287].

**Figure 11:**
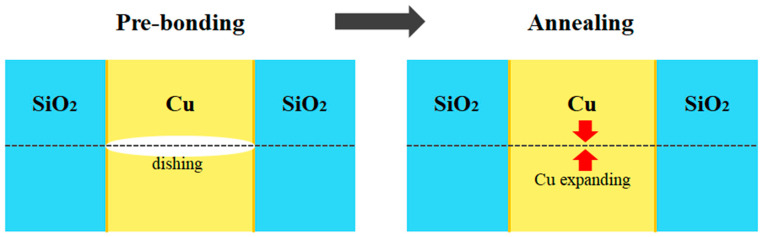
Hybrid bonding flow diagram: Cu dishing control and direct Cu bonding method.

Among them, the Cu-SiO_2_ hybrid bonding method is most likely to develop into a next-generation packaging process. The Cu-SiO_2_ hybrid bonding can generally be formed using a copper dishing control method [288]. This technology is also known as direct Cu-bonding or direct bond interconnect (DBI), which was developed by Ziptronix, Inc. [266,283]. The direct Cu-bonding proceeds through two steps (Figure 11). In the first step, a dielectric-to-dielectric bond is formed at RT, and in the second step, a Cu-to-Cu connection is performed through batch annealing (150–300 °C). Since there is a difference in the CTE of Cu and SiO_2_, copper with a large CTE expands during annealing, contacts each side of the copper, and completes the diffusion bonding. As this process combines the surfaces of the two materials at the atomic level, it is important to have a surface roughness within the order of nanometers and to ensure the bonding surface is clean [284].

Direct Cu-bonding has been used in CMOS devices and appears to be the most suitable process [285,288,289]. Gao et al. recently performed direct Cu-bonding at 200 °C for 1 h using DBI, and the misalignment between the upper and lower Cu-pads was within 5 μm [85]. Additionally, there was no visible gap between the bonding Cu-pads, which indicates a good electrical connection.

Meanwhile, high-temperature conditions can cause damage to the semiconductor chip or deterioration in the quality of the device, so low-temperature bonding is the main point of advanced packaging technology. Many studies are being conducted to achieve hybrid bonding at low and RT [269,284,290,291,292,293,294,295,296,297]. Recently, Liu et al. adopted Pt as a passivation material to achieve low-temperature Cu direct bonding [294]. The Pt passivation layer (10 nm) is used on the Cu surface to prevent Cu oxidation. During the TCB process, Cu atoms diffused across the Pt-bonding interface and facilitated low-temperature bonding. The wafer-level bonding was also successfully conducted at 180 °C for 50 min in a vacuum state, and the pull bonding strength was around 19.7 kgf.

**Figure 12:**
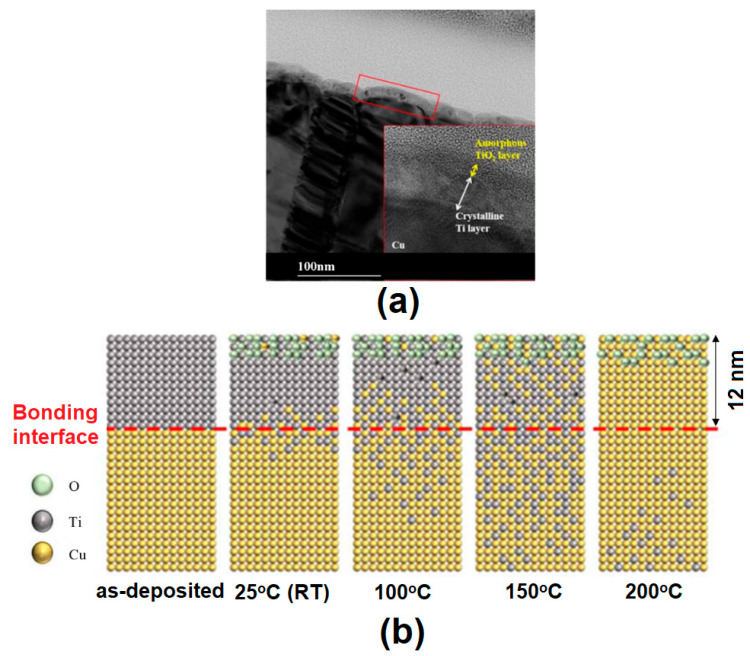
Cu-Cu bonding using a 12 nm thick Ti film. (**a**) TEM image with Ti thin film deposited on Cu surface; (**b**) Schematic diagram of Ti and Cu diffusion behavior [298].

Park et al. deposited a thin film of Ti of about 12 nm on the copper surface to investigate the effect of this on Cu-Cu bonding [298]. In Figure 12a, an amorphous Ti oxide of 3 nm is formed on the surface. Ti and Cu diffused in a low-temperature range between RT and 200 °C, and the diffusion rate of Ti was higher than that of Cu (Figure 12b). Cu-Cu was bonded using a Ti nano-film at 200 °C for 1 h, and the average joint shear strength was 13.2 MPa, which proved the potential of the Ti nano-film.

Cu/SiO_2_ hybrid bonding was successfully performed at 120–150 °C using a metal passivation layer by Liu et al. [297]. For the passivation layer, a 10 nm thickness of Pd or Au was coated on Cu. Without a passivation layer, the Cu direct bonding required a high temperature (>400 °C). The bonded shear strength with passivation layers showed around 60 N and was several times stronger than without a passivation layer.

In order to achieve Cu/dielectric hybrid bonding at RT or 200 °C, He et al. proposed a modified SAB method that combines SAB technology and a prebonding attach-detach process in a vacuum [296]. The SAB method involves irradiating a bonding surface with a Si-containing Ar beam before bonding and bonding it in an ultra-high vacuum (UHV) of 5 × 10^−6^ Pa. The strength of the Cu-Cu joint bonded at RT in UHV was 2.5 J/m^2^, and the strength of the SiO_2_-SiO_2_ joint was 0.5 J/m^2^. Post-bonding treatment at 200 °C for 2 h resulted in no significant change in the bonding strength. Ong et al. demonstrated Cu/SiO_2_ hybrid bonding at 200 °C and 1.06 MPa bonding pressure using (111)-orientation nanotwinned-Cu [280]. The electrical resistance stabilized from 25 to 375 °C and had a smaller contact resistance of 1.2 × 10^−9^ Ω·cm^2^. Accordingly, the nanotwinned-Cu results are promising for low-temperature Cu/SiO_2_ hybrid bonding.

## 8. Reliability of TSV Solder Joints and Future Directions

The joint reliability of Cu-filled TSVs and solder is influenced by the type of solder alloy, bonding temperature, and formation of IMCs at the junction. As discussed, ball mounting, solder injection, and paste printing are just a few of the solder bumping methods that are available for TSVs. Depiver and his co-workers discovered that SAC 405 and SAC 387 had the highest and lowest fatigue lives, respectively [299]. This suggests that even little modifications to the SnAgCu eutectic system composition can have a big impact on dependability. Solder bumps are reflowed to connect stacked TSVs and solder bumps. At the Cu/solder interface, Cu and Sn react in the solid state to form Cu_6_Sn_5_ and Cu_3_Sn IMC at the interface. Reactions (4) and (5) after reflow lead to the production of Cu_6_Sn_5_ [300]:

6Cu + 5Sn → Cu_6_Sn_5_ (4)

2Cu_3_Sn + 3Sn → Cu_6_Sn_5_ (5)

Cu_6_Sn_5_ IMC grows faster when Sn and Cu are diffused in solid form under thermal conditions. Ni atoms replace Cu atoms to generate (Cu, Ni)_6_Sn_5_ IMCs when Ni UBM is used. Finally, Reaction (6) [300] describes the transition of Cu_6_Sn_5_ to Cu_3_Sn at the Cu/Sn interface:

Cu_6_Sn_5_ + 9Cu → Cu_3_Sn (6)

Compared to Cu_6_Sn_5_ IMC, Cu_3_Sn IMC has lower fracture toughness because it is more likely to generate Kirkendall voids. The reliability of the solder junction can be severely impacted by a greater amount of IMC at the bump/Cu contact. Ni, which has a higher activation energy than Cu, interacts with Sn to produce additional Ni_3_Sn_4_ IMCs on Cu pads. As the IMC thickness and reflow conditions are inversely correlated, optimizing the IMC thickness necessitates a low reflow temperature. Moreover, it raises the fraction of IMCs to solder volume when bump size and pitch size are both decreased. Although Sn, Sn-Ag, or SAC305 are soft solders, the interfacial IMCs formed are brittle and decrease the endurance of TSV/solder joints [276].

In a study by Su et al. [301], the dependability of the Cu pillar UBM/solder cap was investigated to ascertain the impacts of solder composition and IMC formation rate. During high-temperature aging, the study discovered that alloy systems, including Sn1Ag, Sn2Ag, and Sn3Ag, behaved differently. During reflow, a Sn1Ag bump showed a significant growth of UBM IMCs (including AuSn_4_ and PdSn_4_), whereas Sn2Ag and Sn3Ag bumps showed a higher fraction of Cu_6_Sn_5_ IMC and fewer UBM IMCs. After high-temperature aging, the IMC thickness grew, and a sizable number of voids were discovered inside Sn1Ag bumps. Cu_3_Sn IMCs were thicker, and there were several Kirkendall voids at the Sn2.0Ag bump interface. In contrast, Sn3.0Ag demonstrated a defectless interface with exceptional reliability.

### 8.1. Nanomodified Solder Bumps

NPs are also added to the solder, which considerably slows down the development of Cu_6_Sn_5_ IMC at the interface. Equation (7) [302] illustrates how the surface energy of the material is reduced and the formation of crystal plane *k* is slowed down when NPs are adsorbed on the IMCs.

(7) $$\sum_{k}\gamma_{c}^{k}A_{k}=\sum_{k}\gamma_{0}^{k}A_{k}-RT\sum_{k}A_{k}\int_{0}^{c}\frac{\Gamma^{k}dc}{c}\;\to maximum$$

Equation illustrates the relationship between the amount of NPs added to the solder and the velocity of crystal plane *k* (7). The surface energy of the crystal plane *k* of IMC with and without NPs are $\gamma_{c}^{k}$ and $\gamma_{0}^{k}$, respectively. Here, c is the concentration of NPs, A_k_ is the area of the plane *k*, and $\Gamma^{k}$ is the number of NPs adsorbed at plane *k*. The first term on the right-hand side is constant for a fixed volume. Thus, the plane $\Gamma^{k}$ will be highly effective in the adsorption of NPs on plane *k.*

Shin et al. prepared the electroplated composite solder bump Sn58Bi reinforced with SiC NPs [25]. A 10 A/dm^2^ current density was used to co-deposit the NPs in the solder matrix. The presence of SiC NPs reduced the distance between the Sn and Bi-layers and the thickness of the Cu_6_Sn_5_ IMC at the solder bump interface. The outcome was an increase in the solder bump’s shear strength of 6% and 10% under the as-reflow and aged conditions, respectively. Solder paste in research frequently contains NPs of ZrO_2_, Al_2_O_3_, ZnO, and CNTs into SAC and Sn-Bi solders to improve mechanical properties and minimize Cu_6_Sn_5_ IMC at the interface [303]. Moreover, it has been demonstrated that adding nanoparticles improves the spreading and wetting properties. For instance, Al_2_O_3_ NPs added to the solder showed higher wettability than monolithic solders [304]. The segregation of NPs within the matrix is a major problem when utilizing solder paste with NPs in fine-pitch applications. The degree of NP addition must be tuned to obtain appropriate wetting and reduced IMC growth. A precise control of the current density and time required for adequate NP dispersion in the solder bump is required in electroplated composite solder bumps.

Chang et al. [305] employed Cu/Sn-solder, Cu/Ni/Sn-solder, and Cu/Ni/Cu/Sn-solder with a pitch <30 µm to investigate the stress distribution and IMCs at the TSV interface. Ni_3_Sn_4_ was demonstrated to form at the solder-Ni-bump interface. Simulation findings showed that a Cu/Ni/Cu/solder structure and Cu/Ni/solder with a thinner Cu thickness of 15 µm effectively reduced thermal stresses at the interface during the thermal cycling test. The Cu/Ni/solder bump showed voids and fractures after five reflows, but the Cu/Ni/Cu/Sn bump was defect-free. The Cu/solder structure displayed a gradual metallic reaction at 175 °C for 300 h, yielding Cu_6_Sn_5_ with a negligible decrease in solder volume and no solder fault. However, at 200 °C and 300 h, the Cu/Ni/Sn solder bump failed, leaving the solder with void and crack flaws. These findings may be connected to the IMC generated at the solder bump contact. According to Chung et al. [306], the Ni_3_Sn_4_ IMC formation rate is quick and results in an 11% reduction of Sn volume, whereas the Cu_6_Sn_5_ IMC reaction layer forms slowly and results in a 5% reduction of Sn volume. Because the dissipation of volume shrinkage primarily takes place in the vertical direction, the development of IMCs may degrade the electrical properties of the TSV/solder joints.

According to Bashir et al. [307], after 400 h of electromigration, Cu_6_Sn_5_ IMC enhanced the electrical resistance of the SAC305/Cu joint. In contrast, (Cu, Ni)_6_Sn_5_ IMC in SAC305/Ni/Cu showed a steady resistance after 600 h. In a different research, SAC 305 electrical resistivity was enhanced from 1.04 × 10^−6^ Ωm to 1.34 × 10^−6^ Ωm by adding 0.03 wt.% CNTs [308]. Controlling processing conditions to reduce IMC formation is essential, given the influence of IMC on the electro-mechanical characteristics of the solder joints. In surface mount technology and flip chip packages, paste mixing is frequently employed to include NPs into the solder bump. Nonetheless, solder bumps for 3D integration technology containing NPs have improved the joint performance as described in [65,284,288] thanks to electroplating.

### 8.2. Emerging Solder Bumps-HEAs

**Figure 13:**
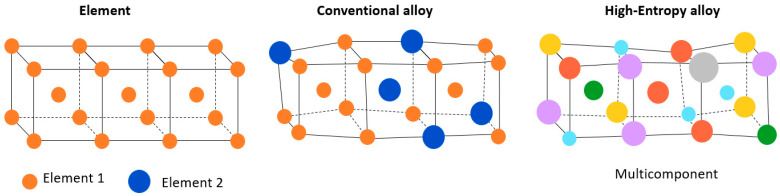
Lattice distortion for elements, conventional alloys, and HEAs [311]. The different color spheres correspond to different alloy components (elements) in the lattice structure.

The creation of novel materials is ongoing to suit the requirements of several sectors, including electronics, petrochemicals, airplanes, cars, and nuclear. Innovative filler metals must be developed to connect these complicated materials since conventional fillers frequently cause the creation of brittle IMCs, element segregation, and thermal stresses at the interface, which can harm joint performance. Because of the low wettability and significant discrepancies in the CTEs of the two materials, connecting metal and ceramic is particularly difficult. HEAs have lately been investigated by researchers as brazing fillers for connecting metal to ceramic to overcome this issue [309,310]. Due to the high entropy effect, HEAs provide better filler element mixing (Figure 13). This improves the metal-to-ceramic connections by causing the production of face-centered cubic (FCC) or body-centered cubic (BCC) structures, preventing the formation of brittle IMCs [311,312].

This section looks at HEAs’ potential as a filler metal for brazing both similar and unrelated materials. As a brazing filler, HEAs have a desired set of characteristics, including excellent wear resistance, strong corrosion and oxidation resistance, and high-temperature stability. The effect of HEAs fillers on the microstructure and mechanical performance of brazing joints is also examined in this paper. Comparative research between HEA fillers and traditional fillers is also included.

Many studies have been conducted on connecting techniques and the use of appropriate fillers for Ni-and Ti-base alloys [313,314]. It is important to remember that Ni-base alloys may not be optimum for joining during fabrication or repair, even if they are created for maximum performance. For instance, solidification cracks in the fusion zone, embrittlement cracks, and heat-affected zone cracks are frequently caused by the fusion bonding of Ni-base alloys due to the segregation of alloying elements and localized stresses. Nevertheless, the use of solid-state diffusion bonding is constrained by the need for high pressure and exact alignment. Brazing, on the other hand, does not need such alignment and operates at a temperature lower than the melting point of the base material, preventing solidification flaws.

The development of multicomponent solders requires extensive research since binary Sn-based solders can no longer keep up with the rapid pace of technological advancement. In the form of HEAs, multicomponent alloys have exceptional qualities, particularly in terms of creep [315], magnetic behavior [316], biocompatibility [317], wear [318], and slow diffusion effect [311]. The use of HEA solder may impart these special characteristics to TSV/solder interconnections, opening a new design space and expanding the uses of solder bumps. According to a recent study, SnBiInZn HEA alloy was used as solder for low-temperature soldering [319]. The solder has a wetting temperature of about 100 °C and a melting point ≈ 80 °C. At the 100 °C reflow temperature, it possesses good wetting qualities and shear strength. Also, the IMC growth kinetics investigation shows that during reflow, a very thin layer of Cu_6_Sn_5_ IMC is formed via a very slow solid-liquid interfacial reaction rate. In this work, HEAs were used as a low melting point solder, an innovative attempt with possible uses in future advanced electronic packaging technology.

## 9. Conclusions and Future Remarks

In this review, we have overviewed the research trajectories for 3D connection—such as fabrication of TSV in Si-wafers and solder bumping—and issues in TSV joining, Cu pillar, low-temperature TLP bonding, and hybrid bonding applicable to 3D TSV stacking have been covered. Three-dimensional TSV electronic packaging technology is emerging as an alternative that can overcome the limitations of semiconductor processing beyond the simple circuit protection and connection functions of semiconductors. In particular, the process for making TSV involves drilling vertical vias on a Si wafer using the DRIE technique, filling the vias with Cu electroplating, and solder bumping on TSV for 3D stacking and integration. The difficulties in TSV fabrication and perfect Cu-filling are discussed in the study, along with concerns with high-density packaging, such as cracking and delamination at the interfaces, insulating layer failure caused by Cu extrusion, IMC formation at joints, and CTE mismatch. In particular, hybrid bonding is evaluated to be a next-generation core back-end process technology to minimize packaging sizes and increase bandwidth. The development of reliable solder bump technology and materials still needs to be studied and improved to increase the electro-mechanical reliability of the TSV filling and bonding technologies described in this review. While the electroplating method for solder bumping is well-established in the industry, more recent innovations like paste printing and HEA solders can help 3D packaging become more affordable. Recent technological advancements in solder bumping are projected to increase package reliability.

The performance of the 3D stacked chips and devices can be tailored only through novel design concepts, package platforms, and new bumping materials with minimum reaction compounds, low power requirements, and superior performance. In the future, it is expected that 3D TSV technologies may replace existing surface mount devices due to their smaller dimension, greater speed, and multi-functional applications.

## Figures and Tables

**Table 1:** Research trends in the development of Cu-filled TSVs and functional layers.

S. No.	TSV Width (µm)	TSV Depth (µm)	AR	Functional Layer	CTE (10^−6^/K)	Ref./Year
1	100	--		SiO_2_/TaN/Cu	0.24/3.6/17.5	[92], 2020
2	13	110	1:8.5	SiO_2_/Ta/Cu	0.24/6.6/17.5	[93], 2020
3	11.5–30.2	92.7–128.1	1:8	SiO_2_/TiN/Cu	0.24/8.1/17.5	[94], 2021
4	9	141	1:15.7	Polyimide/TiN/Cu	40.9/8.1/17.5	[95], 2021
5	3	40	1:13.3	SiO_2_/TiN/Cu	0.24/8.1/17.5	[96], 2021
6	5	40	1:8	Al_2_O_3_/Ti/Pt	8.1/8.5/9.1	[97], 2021
7	10	50	1:5	SiO_2_/Ti/Cu	0.24/8.5/17.5	[98], 2022
8	10	100	1:10	SiO_2_/Ti/Cu	0.24/8.5/17.5	[99], 2022

**Table 2:** Typical parameters for various TSV technologies [115,116].

Parameter	Via (First)	Via (Middle)	Via (Last)
Diameter	4 × 10^−6^ m	4 × 10^−6^ m	10 × 10^−6^ m
Pitch	8 × 10^−6^ m	8 × 10^−6^ m	20 × 10^−6^ m
Length	10 × 10^−6^ m	60 × 10^−6^ m	60 × 10^−6^ m
TSV resistance	5.7 Ω	0.9 Ω	0.02 Ω
TSV inductance	4.2 pH	49.8 pH	34.9 pH
TSV coupling capacitance	1.2 fF	6.7 fF	6 fF

**Table 3:** Comparison of activation energies for the IMC formation between solder and Cu from previous studies.

Joint Couple	Method	Aging Temperature (°C)	Q (kJ/mol)	Ref, Year
Sn-16 wt.% Sb/Cu	Dip soldering	120–170	68.27	[236], 2019
Sn-58 wt.% Bi	Laser soldering	85–115	115.6	[237], 2021
Sn-58 wt.% Bi-2 wt.% Ag	Laser soldering	85–115	138	[237], 2021
Cu41 wt.% Sn11 wt.%/Cu	TLP bonding	390–450	108.19	[238], 2022
Sn-Bi-In-Ag/Cu	Reflow soldering	120–160	10.4	[239], 2020
Sn-Bi-In/Cu	Reflow soldering	120–160	11.1	[239], 2020
Sn-0.075 wt.% CNT/Cu	Reflow soldering	130–170	58.19	[240], 2021
Sn-4 wt.% Ag-0.5 wt.% Cu/Cu	Reflow soldering	150–175	65.6	[241], 2021
Sn-4 wt.% Ag-0.5 wt.% Cu + 3 wt.% Bi + 0.05 wt.% Ni/Cu	Reflow soldering	150–175	81.9	[241], 2021
Sn-58 wt.% Bi/Cu	Reflow soldering	100–200	48.22	[242], 2021
Sn-58 wt.% Bi-0.1 wt.% Ti/Cu	Reflow soldering	180–200	58.2	[242], 2021
Sn-3.5 wt.% Ag/Cu	Reflow soldering	100–180	65.9	[233], 2022
Sn-3.5 wt.% Ag-0.007 wt.% graphene/Cu	Reflow soldering	100–180	77.0	[233], 2022
Sn-37 wt.% Pb/AuNi plated Kovar	Reflow soldering	120–170	95.96	[243], 2019
Sn-3 wt.% Ag-0.5 wt.% Cu/Cu	Reflow soldering	100–150	57.71	[234], 2022
Sn-3 wt.% Ag-0.5 wt.% Cu-0.1 wt.% TiO_2_ NPs/Cu	Reflow soldering	100–150	69.32	[234], 2022
Sn-3 wt.% Ag-0.5 wt.% Cu/Cu	Reflow soldering	85–150	52	[244], 2021
Sn-3 wt.% Ag-0.5 wt.% Cu-1 wt.% Kaolin/Cu	Reflow soldering	85–150	74	[244], 2021

**Table 4:** Solidus, liquidus, and solidification duration of important Sn-Bi-X solders.

Sn-Bi Composition	Solidus (°C)	Liquidus (°C)	Solidification Time (°C)	Ref.
Sn-58 wt.% Bi	130.2	139	8.8	[254]
Sn-58 wt.% Bi	136.1	139.1	3	[255]
Sn-58 wt.% Bi	136.1	139.1	3	[256]
Sn-58 wt.% Bi	138.3	139.3	1	[257]
Sn-58 wt.% Bi	139.3	147.6	8.3	[258]
Sn-58 wt.% Bi	139.5	147.6	8.1	[259]
Sn-58 wt.% Bi	139.4	148.0	8.6	[260]
Sn-58 wt.% Bi	139.6	147.4	7.8	[261]
Sn-58 wt.% Bi	140.3	146.0	5.7	[262]
Sn-58 wt.% Bi	138	144	6	[263]
Sn-45 wt.% Bi	138	155	17	[263]
Sn-40 wt.% Bi	138	168	30	[263]
Sn-35 wt.% Bi	138	178	40	[263]
Sn-55 wt.% Bi	138	186	48	[263]
Sn-58 wt.% Bi-0.5 wt.% Ti	138.9	142.7	3.8	[264]
Sn-58 wt.% Bi-1 wt.% Ti	139.1	143.4	4.3	[264]
Sn-58 wt.% Bi-0.1 wt.% Ag	136.2	139.7	3.5	[255]
Sn-58 wt.% Bi-0.5 wt.% Ag	135.7	138.2	2.5	[264]
Sn-58 wt.% Bi-0.5 wt.% Ag-0.1 wt.% Ce	136.6	139.1	2.5	[255]
Sn-58 wt.% Bi-0.5 wt.% Ag-0.1 wt.% La	136.6	139.1	2.5	[255]
Sn-58 wt.% Bi-1 wt.% Ag	137	142	5	[264]
Sn-58 wt.% Bi-1 wt.% Ag-1 wt.% In	133	137	4	[264]
Sn-58 wt.% Bi-1 wt.% Ag-3 wt.% In	125	133	8	[264]
Sn-58 wt.% Bi-2 wt.% Ag	139.1	145.4	6.3	[261]
Sn-58 wt.% Bi-2 wt.% Ag-2 wt.% In	131.2	136	4.8	[261]
Sn-58 wt.% Bi-2 wt.% In	129.8	135	5.2	[261]
Sn-58 wt.% Bi-3 wt.% In	119.9	140.5	20.6	[258]
Sn-58 wt.% Bi-0.05 wt.% Co	140.3	147	6.7	[262]
Sn-58 wt.% Bi-0.5 wt.% Co	140.1	145	4.9	[262]
Sn-40 wt.% Bi-0.1 wt.% Cu	125.1	132.2	7.1	[254]
Sn-40 wt.% Bi-2 wt.% Zn-0.1 wt.% Cu	127.7	136.3	8.6	[254]
Sn-52 wt.% Bi-1.8 wt.% Sb	140.6	152.0	11.4	[260]
Sn-44 wt.% Bi-1.8 wt.% Sb	141.9	180.5	38.6	[260]
Sn-48 wt.% Bi-1 wt.% Sb	140.6	168.7	28.1	[260]
Sn-48 wt.% Bi-2 wt.% Sb	142.3	169.7	27.4	[260]

## Data Availability

Not applicable.
